# Genomic Tools in Biological Invasions: Current State and Future Frontiers

**DOI:** 10.1093/gbe/evad230

**Published:** 2023-12-18

**Authors:** Angela McGaughran, Manpreet K Dhami, Elahe Parvizi, Amy L Vaughan, Dianne M Gleeson, Kathryn A Hodgins, Lee A Rollins, Carolyn K Tepolt, Kathryn G Turner, Kamolphat Atsawawaranunt, Paul Battlay, Carlos Congrains, Angelica Crottini, Tristan P W Dennis, Claudia Lange, Xiaoyue P Liu, Paige Matheson, Henry L North, Iva Popovic, Marc Rius, Anna W Santure, Katarina C Stuart, Hui Zhen Tan, Cui Wang, Jonathan Wilson

**Affiliations:** Te Aka Mātuatua/School of Science, University of Waikato, Hamilton, New Zealand; Biocontrol and Molecular Ecology, Manaaki Whenua Landcare Research, Lincoln, New Zealand; School of Biological Sciences, Waipapa Taumata Rau/University of Auckland, Auckland, New Zealand; Te Aka Mātuatua/School of Science, University of Waikato, Hamilton, New Zealand; Biocontrol and Molecular Ecology, Manaaki Whenua Landcare Research, Lincoln, New Zealand; Centre for Conservation Ecology and Genomics, Faculty of Science and Technology, University of Canberra, Canberra, ACT, Australia; School of Biological Sciences, Monash University, Melbourne, VIC, Australia; Evolution and Ecology Research Centre, University of New South Wales, Sydney, NSW, Australia; Department of Biology, Woods Hole Oceanographic Institution, Woods Hole, MA, USA; Department of Biological Sciences, Idaho State University, Pocatello, ID, USA; School of Biological Sciences, Waipapa Taumata Rau/University of Auckland, Auckland, New Zealand; School of Biological Sciences, Monash University, Melbourne, VIC, Australia; Entomology Section, Department of Plant and Environmental Protection Sciences, University of Hawaiʻi at Mānoa, Honolulu, HI 96822, USA; US Department of Agriculture-Agricultural Research Service, Daniel K. Inouye US Pacific Basin Agricultural Research Center, Hilo, HI 96720, USA; CIBIO, Centro de Investigação em Biodiversidade e Recursos Genéticos, InBIO Laboratório Associado, Campus de Vairão, Universidade do Porto, Vairão 4485-661, Portugal; Departamento de Biologia, Faculdade de Ciências, Universidade do Porto, Porto 4169–007, Portugal; BIOPOLIS Program in Genomics, Biodiversity and Land Planning, CIBIO, Vairão 4485-661, Portugal; Department of Vector Biology, Liverpool School of Tropical Medicine, Liverpool, UK; Biocontrol and Molecular Ecology, Manaaki Whenua Landcare Research, Lincoln, New Zealand; Department of Marine Science, University of Otago, Dunedin, New Zealand; Te Aka Mātuatua/School of Science, University of Waikato, Hamilton, New Zealand; Department of Zoology, University of Cambridge, Cambridge, UK; School of the Environment, University of Queensland, Brisbane, QLD, Australia; Centre for Advanced Studies of Blanes (CEAB, CSIC), Accés a la Cala Sant Francesc, Blanes, Spain; Department of Zoology, Centre for Ecological Genomics and Wildlife Conservation, University of Johannesburg, Johannesburg 2006, South Africa; School of Biological Sciences, Waipapa Taumata Rau/University of Auckland, Auckland, New Zealand; School of Biological Sciences, Waipapa Taumata Rau/University of Auckland, Auckland, New Zealand; School of Biological Sciences, Waipapa Taumata Rau/University of Auckland, Auckland, New Zealand; The Organismal and Evolutionary Biology Research Programme, University of Helsinki, Helsinki, Finland; School of Biological Sciences, Monash University, Melbourne, VIC, Australia

**Keywords:** biological invasion, invasion genomics, invasive species, pest, management

## Abstract

Human activities are accelerating rates of biological invasions and climate-driven range expansions globally, yet we understand little of how genomic processes facilitate the invasion process. Although most of the literature has focused on underlying phenotypic correlates of invasiveness, advances in genomic technologies are showing a strong link between genomic variation and invasion success. Here, we consider the ability of genomic tools and technologies to (i) inform mechanistic understanding of biological invasions and (ii) solve real-world issues in predicting and managing biological invasions. For both, we examine the current state of the field and discuss how genomics can be leveraged in the future. In addition, we make recommendations pertinent to broader research issues, such as data sovereignty, metadata standards, collaboration, and science communication best practices that will require concerted efforts from the global invasion genomics community.

**Significance**

Invasion genomics is a rapidly advancing field that aims to answer questions about the genetic mechanisms underlying biological invasion. Exciting new developments are enabling better detection of new incursions, rapid identification of invasion routes, and more comprehensive understanding of adaptive processes during invasion. This work holds promise for future developments, including better prediction of invasive potential and more advanced surveillance and mitigation approaches that take a genome-informed view. In this review, we discuss the capacity of genomic tools to inform our current understanding of biological invasion, the next frontier of questions that genomic tools are poised to help us answer, and the recommendations pertinent to data management and dissemination that must accompany this progress.

## Introduction

Invasive species are a major threat to biodiversity, global food security, and livelihoods. Costs of managing invasive species are growing globally, with emerging economies the most vulnerable. As our planet experiences warming punctuated by more frequent severe weather events and ever-increasing connectivity from trade and transport, global rates of biological invasion are predicted to continue to rise, compounding the impact of invasive species on already stressed ecosystems (Essl et al. 2020).

After decades of research, we still do not fully understand how invasive species successfully colonize new habitats despite being exposed to potentially novel environmental and ecological challenges (Novoa et al. 2020). Though the importance of phenotypic characteristics (e.g. dispersal capacity, rapid reproduction, phenotypic plasticity, and behavioral thermoregulation) and ecological factors (e.g. competition) is well recognized (Colautti et al. 2017; Jardeleza et al. 2022), new research is providing evidence that genomic data are rich with information relating to invasion success (e.g. Makino and Kawata 2019; Olazcuaga et al. 2020).

**Fig. 1.**
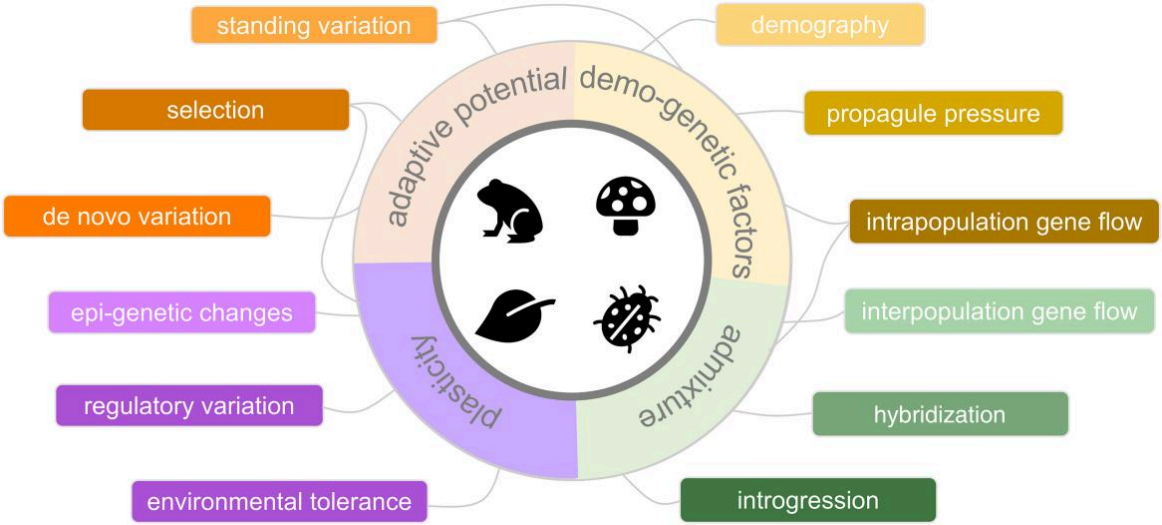
Conceptual diagram capturing various evolutionary factors and processes involved in biological invasions. The strength of some factors/processes is likely to vary depending on the invasion stage (i.e. preintroduction, transport, introduction, establishment, and spread). Lines represent overlap among different processes, which should be considered in unison when examining genetic patterns within invasive species.

**Fig. 2.**
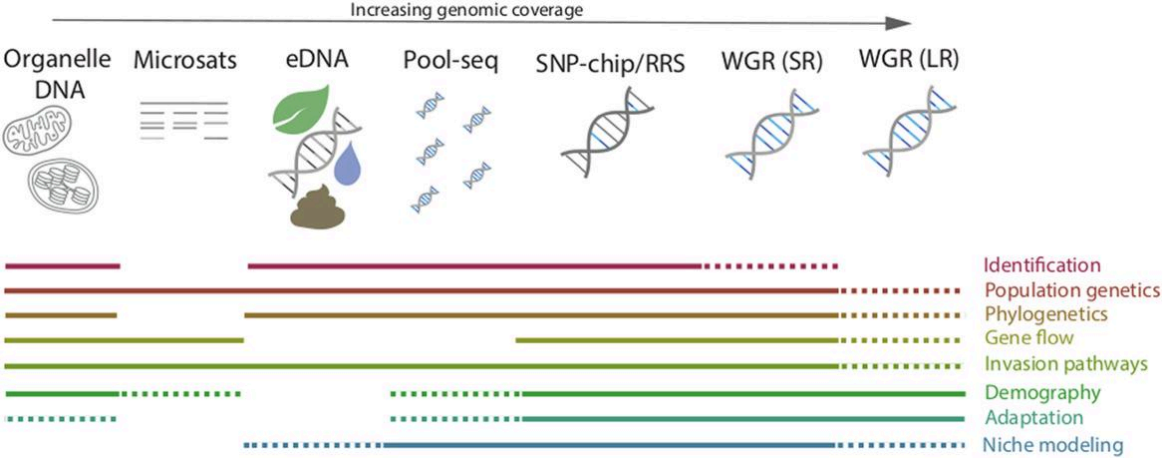
Summary of genomic data types and their applications in invasion genomics. High-throughput sequencing data have begun to replace traditional genetic markers (e.g. organelle sequences or microsatellite markers) in the study of invasive species, with the figure demonstrating increasing genomic coverage across a range of genetic/genomic data types. Among high-throughput methods are environmental DNA (eDNA) and Pool-seq, single nucleotide polymorphism (SNP)-chip/reduced-representation sequencing, and whole genome resequencing (WGR) approaches for both short- and long-read data (SR and LR, respectively). While some types of genomic data are useful across a broad range of applications in invasion genomics, others have a more narrow application, as indicated by the colored lines. For example, eDNA and Pool-seq data, while useful for species identification and population genetics analysis, lack the individual sample level resolution needed for some other analysis types; and many data types may be used in niche modeling (or species distribution modeling), though microsatelites typically lack the resolution necessary for this type of analysis and others. Dashed lines indicate situationally useful/necessary data; for example WGR (LR) is ideal for population genetic studies looking at complex variants but may not be needed otherwise, and organelle DNA can be assessed for putative patterns of adaptation but lacks the genome-wide variant view afforded by sequencing technologies with higher genomic coverage.

Of active interest is the use of genomic approaches to investigate mechanistic questions, such as whether genomic variation that facilitates invasion is already present in the native range before invasion (Eyer et al. 2018; Sherpa et al. 2019), where it may provide a substrate for selection in the new environment (Exposito-Alonso et al. 2018; Tepolt et al. 2022; Battlay et al. 2023) (Fig. 1). Genomic data are illuminating our understanding of the types and tempos of adaptation during biological invasions, as well as how demography and rapid adaptation intersect to shape genetic variation in introduced populations. Another emergent area is the use of genomic technologies to rapidly detect incursions and their pathways into new environments (Fig. 2). In particular, genomic data offer a high-throughput and time-efficient complementary approach to biosecurity surveillance and diagnostics (Trujillo-González et al. 2022).

In this review, we examine the current state of invasion genomics research. Our aim is to evaluate how genomic tools are currently being used to identify, understand, and manage biological invasions and how technological advances may change the way this is done in the future. Collating expertise from international experts who recently participated in the inaugural conference on Invasion Genomics (November 2022; Aotearoa New Zealand; https://www.invasomics.com/conferences), we highlight important research questions and draw attention to key data, collaboration, and communication standards that will benefit the field moving forward. In the first section, we review research that identifies genomic signatures underlying invasion success. In the next section, we consider how well we are placed to predict invasive potential and translate findings from invasion genomics to other fields. The third section focuses on how technology is transforming our ability to identify and detect invasive species in large-scale surveillance approaches. Finally, the fourth section reflects on the next frontier in invasion genomics, identifying key outstanding questions upon which we might best focus collective resources. Throughout this review, we define invasive species as those that reach new habitats outside their native range, spread from their point of introduction, and cause detrimental impacts on environments and economies in the introduced range.

## Genomic Signatures

An increasing number of studies have identified a key role for standing genetic variation in providing the substrate for postinvasion adaptation as a species establishes and spreads in a new environment (Vandepitte et al. 2014; Tepolt et al. 2022; Battlay et al. 2023). For example, certain alleles may be maintained through balancing selection in the native range but experience directional selection in novel habitats (Stern and Lee 2020). Similarly, adaptive differences across an extensive native range may mean that some native populations are more “preadapted” than others to the challenges posed by different invadable environments (Rius et al. 2015). Introduced populations often experience founder effects because of demographic bottlenecks that decrease genetic diversity and can impact invasion success. However, despite this “genetic paradox of invasion,” many invasive species do retain high diversity when measured at appropriate genetic markers, while others thrive due to other aspects, such as multiple introductions, phenotypic plasticity, asexual reproduction, and hybridization (Estoup et al. 2016).

Postintroduction adaptation may also be facilitated by beneficial de novo mutations that arise in the introduced range and are rapidly selected (Exposito-Alonso et al. 2018). Such mutations may compound fitness advantages with organismal plasticity, which may itself be preadapted or evolve in the new range (Zenni et al. 2014). For example, high plasticity in combination with rapid genetic adaptation explains the successful invasion of the plantain *Plantago virginica* in novel environments that are often heavily managed and nitrogen enriched (Luo et al. 2019).

Hybridization, introgression (between individuals or species or due to multiple introductions from genetically diverse source populations), and polyploidy can also facilitate invasion by mixing divergent sources of genomic variation that have evolved in different environments, allowing the introduced population to draw on a deeper pool of adaptive diversity than any of its parents (Makino and Kawata 2019; Popovic et al. 2021). Conversely, maladaptation through genetic swamping from environmentally different habitats may impede species from entering parts of the introduced range that are not environmentally similar to the native range (Polechová and Barton 2015).

Since many invasive species are repeatedly introduced and spread in multiple distinct regions of the globe, we have the opportunity to use these introductions as replicated natural experiments. Among other things, this replication can help determine how genome characteristics alongside evolutionary processes, such as gene flow and adaptation, can facilitate invasion success. In this section, we explore how genomic data can be used to identify invasive signatures.

### Gene Flow

Successful invasive species are often characterized by high gene flow (Gaither et al. 2013). Gene flow is predicted to promote balanced polymorphism, especially for alleles of large effect (Yeaman 2013), and may also generate standing genetic variation at such loci, upon which selection can then act in the introduced range (Tepolt et al. 2022; Battlay et al. 2023).

Increasing episodes of human-mediated transport of species increases opportunities for intraspecific gene flow, as well as secondary contact and hybridization between native and introduced species. If hybrids reproduce and/or backcross, this will result in a mosaic pattern of ancestry along chromosomes (i.e. admixture) as specific genetic variants move from one lineage into another (i.e. introgression; Le Moan et al. 2021). Detecting and quantifying these phenomena is central to a range of proactive and reactive genomic monitoring strategies, though the demographic complexity of biological invasions means that this is rarely straightforward. First, genomic data can be used to reconstruct invasion histories to understand when and where incursions have occurred (Cristescu 2015). Notably, admixture in “bridgehead” populations (when invasive populations are themselves the source of additional new introductions) may alleviate the deleterious effects of a population bottleneck by increasing heterozygosity (Estoup et al. 2016). Second, admixture can be used to quantify gene flow within established metapopulations (Marcus et al. 2021), helping to determine the likelihood that adaptive variants will spread (e.g. pesticide resistance alleles) and the probability of recolonization after local eradication (Paris et al. 2023). Third, population-genomic approaches are important for quantifying otherwise cryptic evolutionary processes, such as adaptive introgression between closely related native and invasive species or demographic swamping of native species (Valencia-Montoya et al. 2020).

When invasion events are analyzed using genomic data, it is possible to identify the presence of hybrids between divergent populations or species (Chakraborty and Rannala 2023), as well as contemporary gene flow (Wilson and Rannala 2003). If intraspecific genetic admixture is sufficiently recent (i.e. introgressed genomic segments are long and intact) and large numbers of loci are sampled, hybrids can be identified with heuristic clustering approaches that estimate the individual ancestry proportions inherited from two or more known source populations (Alexander et al. 2009; Frichot et al. 2014). Once parental and hybrid populations are established, further information on the timing and direction of admixture, and the influence of selection on genomic introgression patterns, can be obtained (Leitwein et al. 2020). For example, genomic data revealed that invasive populations of the brown anole (*Anolis sagrei*) have hybridized with native-range lineages due to changes in natural selection during invasion, emphasizing that changes in selection pressures can facilitate hybridization in the introduced range (Bock et al. 2021).

### Adaptive Evolution

The capacity of a species to adapt to novel abiotic (e.g. climate variability) and biotic (e.g. competition or escape from predators) conditions can be critical to a successful invasion event, and genomic signals of selection may be detected using numerous approaches (reviewed in Hoban et al. 2016). For example, analyses based on identifying outlier alleles with high levels of genetic differentiation (e.g. Olazcuaga et al. 2020) or on those associated with environmental variables, such as topography or climate (e.g. Rellstab et al. 2015), have identified a number of candidate genes or alleles for adaptive change during invasions in animals (e.g. genes important for growth, development, bioenergetics, Yin et al. 2021; detoxification and olfaction, Parvizi et al. 2023) and plants (e.g. genes associated with abiotic stress and pathogen attack, Li et al. 2022; and flowering time, Bieker et al. 2022; Battlay et al. 2023).

Similar evolutionary outcomes can result from both existing variation (allowing for more rapid allele frequency changes postintroduction; Battlay et al. 2023) and novel mutations (Exposito-Alonso et al. 2018), and both play important roles in the response of invasive species to the selective pressure of new conditions. For example, insecticide resistance can evolve from standing genetic variation via selection on a range of genes (e.g. in populations of Colorado potato beetle; Pélissié et al. 2021) and can be passed from an invasive to a native species by adaptive introgression (e.g. in cotton bollworm, *Helicoverpa armigera*; Valencia-Montoya et al. 2020). Thus, different underlying adaptive mechanisms can drive convergent phenotypic responses in different lineages/taxa exposed to similar selective pressures (Pélissié et al. 2021).

The sudden, dramatic environmental shifts experienced by invasive species upon entering the introduced range may also result in adaptive genetic architectures that include large-effect variants (Orr 1998). Such variants include deletions, duplications, insertions, or inversions and are expected to be useful in the maintenance of local adaptation in the face of gene flow (Yeaman and Whitlock 2011)—a common situation for species expanding across diverse climatic gradients. For example, structural variants are increasingly discovered in nonmodel organisms as our ability to assemble high-quality reference genomes, resequence samples to high depths of coverage, and use long-read sequencing technologies increases (Chaisson et al. 2019). Emergent methods (e.g. Li and Ralph 2019) are also increasing understanding of how large-effect variants may facilitate invasion processes. For example, transposable element insertions (e.g. Stapley et al. 2015) and chromosomal inversions (e.g. Tepolt et al. 2022; Battlay et al. 2023) have each been shown to alter gene action to promote adaptation during invasions.

### Plasticity

Besides genetic variation and rapid adaptation, plasticity can alter phenotypes in response to environmental change and may itself be a target of selection (reviewed in Ghalambor et al. 2007). A key mechanism underlying phenotypic plasticity is epigenetic change—the reprogramming of gene expression caused by chemical DNA alteration, histone modifications, and some RNA types (Skvortsova et al. 2018). Such changes do not modify the genome directly but may be heritable, and conditions that invasive species experience in the early stages of colonization may activate epigenetic mechanisms as part of an adaptive response (reviewed in Marin et al. 2020). Notably, epigenetic diversity was found to be inversely correlated with genetic variation at the population level during independent introductions of the house sparrow (*Passer domesticus*) to Africa (Liebel et al. 2013), suggesting that epigenetic changes may in some cases compensate for the decreased genetic diversity that often characterizes biological invasion (Schrey et al. 2012).

### Museomics

**Fig. 3.**
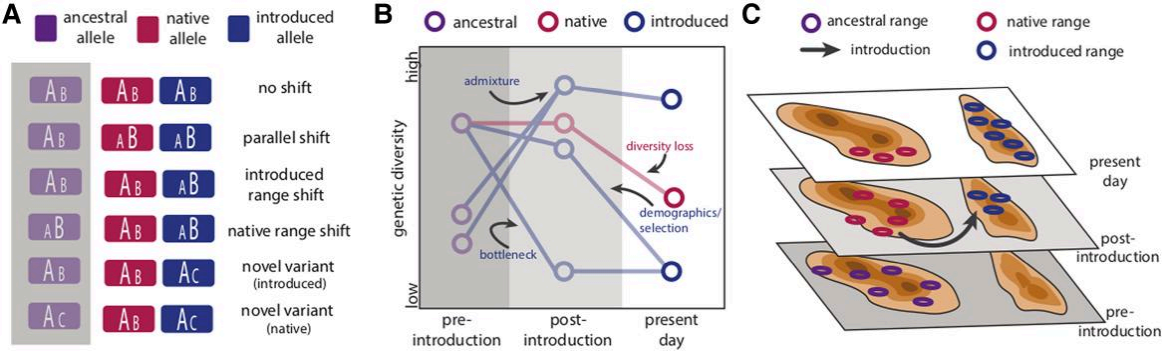
The historical context of invasions. Historical samples can clarify mechanisms of invasion (e.g. preinvasion and postinvasion adaptive dynamics and connectivity across time and space) by aiding A) adaptation studies, as allele state information (ancestral, native, and introduced) is crucial for inferring changes in allele frequency and novel mutations in the native and introduced range (size of alleles A and B indicates relative frequency within each population); B) diversity studies, as it can help contextualize shifts in genetic diversity within both native and introduced ranges; and C) range modeling and landscape genomics studies to contextualize present day invasive species distributions and/or allele frequency correlates with environmental gradients.

As the cost of sequencing continues to fall and historical DNA techniques are refined, the inclusion of temporally resolved data from museum specimens in population genomic datasets, i.e. “museomics”, has become tractable. Tracking DNA changes in populations over time allows for the most direct tests of adaptation using genomics, and a growing number of evolutionary genomic studies are using natural history collections of introduced species to capture temporal change (Vandepitte et al. 2014; Exposito-Alonso et al. 2018; Alves et al. 2019; Bieker et al. 2020; Stuart et al. 2022; Battlay et al. 2023). In the context of biological invasions, such studies can provide key insights into the ability of species to cope with rapid environmental change (Fig. 3) and enable the study of convergent evolution across invasions.

Genomic analyses of historical samples demonstrate a key role for rapid adaptation following introduction to a new range (Vandepitte et al. 2014; Battlay et al. 2023). One of the earliest historic genomic studies to identify such patterns made use of RADseq data in Austrian Rocket (*Sisymbrium austriacum* subsp. *chrysanthum*; Vandepitte et al. 2014). Large allele frequency changes in flowering time genes were identified—even in the earliest herbarium samples—likely reflecting adaptation from standing variation during the initial stages of the introduction. Similarly, in common ragweed (*Ambrosia artemisiifolia*), chromosomal inversions introduced from the native range showed dramatic frequency shifts in the introduced range over time (Battlay et al. 2023). By contrast, in self-fertilizing thale cress (*Arabidopsis thaliana*), genome sequencing of herbarium and modern specimens identified de novo mutations that had risen to intermediate or high frequencies in the North American lineage, a subset of which are associated with locally adaptive traits (Exposito-Alonso et al. 2018).

Temporal data offer insight into the strength and speed of selection over the course of an invasion. The sequencing of natural history collections can allow selection coefficients to be estimated over time scales typically inaccessible to most evolutionary studies in the field (Alves et al. 2019; Battlay et al. 2023). Further, changes in the strength of selection over the course of invasion can be captured. For example, exome sequencing of European rabbits (*Oryctolagus cuniculus*) in France, the UK, and Australia identified strong selection on standing variants following the release of the myxoma virus in Europe and Australia. However, as the virulence of the virus waned, the strength of selection on those variants declined (Alves et al. 2019). In common ragweed, estimates of the strength of spatially varying selection along a latitudinal cline were strongly related to the extent of temporal change in those same loci, demonstrating that the tempo of adaptation was influenced by the strength of local selection (Battlay et al. 2023).

Historical samples can provide insights into the demographic history of invaders and their source populations (e.g. Bieker et al. 2022). Sequencing historical samples may also shed light on the genetic costs associated with demographic changes experienced during introduction. For example, under certain circumstances, genetic load is expected to accumulate during range expansion or genetic bottlenecks (Hodgins et al. 2018). Thus, museum specimens can provide empirical examples of the relationship between range expansion, population size changes, and genetic load.

Metagenomic data gleaned from natural history collections offer opportunities to study ecological mechanisms contributing to invasion success, particularly with regard to microbial interactions. For example, the differential association of microbial pathogens (*Xanthomonas* spp.) combined with evidence of rapid adaptation in the common ragweed host—both derived from historic genome sequencing—potentially underpin its exceptional invasion success across Europe (Bieker et al. 2022).

## Identifying Future Threats

In this section, we review how genomic data can be used for improved prediction and management of future biological invasions.

### Predicting Invasion Potential

An important consideration in the prediction of invasive potential is that we typically only study successful invasions, which may bias our understanding. Though it is difficult to study failed invasions, research into the causes of failed biocontrol and studies of historical data (Marsico et al. 2010) suggest that invasion success or failure often hinges on species traits. For example, of 2,760 intentional avian introductions, birds that reproduced several times a year and had a short lifespan, or those that reproduced once per year but had a long lifespan, were more likely to successfully establish (Sol et al. 2012). However, genomic characteristics are also likely to be important, particularly since invasion dynamics are context and species dependent (Ni et al. 2021).

Utilizing omic data sets for predicting invasive potential and forecasting invasion pathways is a primary objective of invasion genomics. One method involves the use of genotype–environment associations in tandem with climate and land-use change projections to associate habitat distribution with genomic variation as environments shift. Current species niche distribution models for biological invaders are sometimes informed by genomics (e.g. Sillero et al. 2020), and gradient forest analysis is a promising tool that uses machine learning to iteratively partition climatic variables and adaptive genetic variation to explain allele frequency variance in expanding niches (Fitzpatrick and Keller 2015). Utilizing such methods creates a putative pathway not only to infer where invasions originate but also to predict where they may expand.

Evaluating symbiotic microbes in risk analyses and spread modeling is a promising approach for determining invasive potential. An organism's holobiont (i.e. itself and its network of closely associated organisms, including microbes) has the potential to affect invasion success. For example, microbial symbionts of insects provide essential functions and coevolve with their hosts, and can facilitate invasions by providing detoxification capabilities and/or aiding host range expansion (Lange et al. 2023). Similarly, the absence or presence of plant endophytes can determine if a plant becomes invasive (Kentjens et al. 2023), and rapid adaptation and dispersal have led to spatial variation in the resistance of the cane toad (*Rhinella marina*) to its native-range lungworm parasite (*Rhabdias pseudosphaerocephala*) (Eyck et al. 2022).

Finally, comparative interspecific and intraspecific genomic approaches in conjunction with high-resolution spatiotemporal metadata can extend our existing mechanistic understanding toward predictive applications (including regarding adaptation; see Genomic Signatures). Recent studies have attempted to identify globally relevant invasive genomic traits within species, with promising results across a few taxa (e.g. brown marmorated stink bug, *Halyomorpha halys*, Parvizi et al. 2023; fall armyworm, *Spodoptera frugiperda*, Chen et al. 2023). However, a lack of well-curated metadata for invasive species genomic resources has stymied the translation of these trends to a general predictive tool (Vaughan et al. 2023).

### Translation of Invasion Genomics to Other Fields

Invasive species present a framework to understand how environmental change affects the evolution of all species. In a fast-changing world, investigations into the adaptive potential of invasive species can thus help inform understanding of adaptive mechanisms in other species that may be required to rapidly evolve.

Understanding how small populations persist in an invasion scenario may be translatable to conservation practices (Seaborn et al. 2021). For example, a common goal of restoration programs is the establishment of a self-sustaining population, which typically requires a large, genetically diverse group of individuals from multiple source populations over repeated introduction events. This procedure represents a common invasion scenario relevant to conservation, where the invading/threatened population may benefit from introgression/genetic rescue (Todesco et al. 2016). Examples from self-sustaining introductions may help inform the choice of appropriate source populations for facilitated conservation management (often not straightforward; Lovell et al. 2021), including the necessary levels of genetic and population diversity required for successful translocation efforts. Further, learning from successfully established invasive populations may inform the potential loss of local adaptation and unique genetic diversity in threatened populations (Turner et al. 2018).

Invasion genomics provides a mechanistic understanding of how organisms rapidly adapt to novel environments (see Genomic Signatures); the development of biological controls can benefit greatly from such evolutionary perspectives (Fagan et al. 2002). For example, in the harlequin ladybird (*Harmonia axyridis*), postintroduction admixture of some populations and evolutionary changes following a bridgehead event enabled range expansions well beyond what was predicted from range and host testing (Facon et al. 2011). In fact, this biocontrol agent eventually became an invasive species, thus illustrating the fine line between biocontrol and invasion scenarios and the potential for synergistic lessons from both fields.

New ideas are emerging in the field of epidemiology and vaccine development that are benefiting from a strengthened understanding of the evolutionary mechanisms underpinning biological invasion. For example, SARS-Cov-2 evolution demonstrated invasion-typical features: strong functional selection (Bloom et al. 2023), rapid and multilineage adaptation (Markov et al. 2023), and initial admixture in the putative ancestral lineages (Haddad et al. 2021)—all contributing to its rapid expansion across the human population. Given that pathogens and vaccines are caught in a perpetual evolutionary arms race, vaccine development is also benefiting from deeper mechanistic understanding of pathogen invasion and subsequent evolutionary change (Nuismer and Bull 2020).

## Technologies and Applications in Invasion Genomics

In this section, we shift focus toward the ways in which emergent genome-sequencing technologies are facilitating better detection, biosurveillance, and mitigation approaches.

### Detection and Biosurveillance

Genomic data can be used to rapidly identify and track invasive species in a particular environment or via certain trade pathways (Westfall et al. 2020), including those difficult to detect using traditional methods, such as microorganisms, small invertebrates, and cryptic species. Recent studies have demonstrated the power of genomic tools for invasive species detection—even from trace amounts of DNA. For example, Trujillo-González et al. (2022) extracted environmental DNA (eDNA) from airborne and floor dust samples in shipping containers and detected invasive khapra beetles (*Trogoderma granarium*) in Australia, while Dhami et al. (2016) also used quantitative polymerase chain reaction (qPCR) to identify various invasive fruit flies in Aotearoa New Zealand from empty pupal cases or eggs—samples from which diagnostics via traditional methods are often not possible. Recent advances in genome sequencing have also facilitated multilocus approaches for diagnostics, which have provided the increased resolution needed for more precise identification of species, particularly for those groups of species where using single-locus markers has been challenging (e.g. fungi; Dupis et al. 2012).

Genome-sequencing technologies have also enabled rapid generation and assembly of complex genomes of nonmodel species, including many invasive species (but see Matheson and McGaughran 2022). Genome-skimming approaches that generate between 0.1× and 1× coverage of an organism's genome enable cost-effective development of genome-based tools for biosurveillance. Novel biosurveillance tools arising from developments in genome-editing technology (e.g. CRISPR-Cas) are also being deployed for sensitive single-species detection applications, especially where assay development (e.g. qPCR) may be difficult (Williams et al. 2021).

Beyond the identification of individual species, metabarcoding can be used to identify and quantify multiple species in a complex biological community (David et al. 2021), including those that travel together to become invasive (e.g. in Southern bull kelp, *Durvillaea antarctica*, whole communities of invertebrates travel in the holdfast of the kelp and have reached distant Antarctic shores; Fraser et al. 2022). Metabarcoding goes further than the detection of invasive species to provide an understanding of how invaders impact an ecosystem. For example, by comparing species compositions (and potentially relative abundances) in invaded and uninvaded ecosystems, researchers can identify the species that are most affected by invasion and the ecological processes that are disrupted (Dufresnes et al. 2019). Metabarcoding can also be used to guide adaptive management strategies by sampling before and after management interventions to determine their effectiveness in restoring biodiversity and ecosystem function (i.e. whether the abundance of invasive species has been reduced and/or whether native species have been restored).

### Management of Invasive Species

Establishing the origin of invasion pathways may assist biosecurity efforts by facilitating control of movement across invasion routes (Pichlmueller et al. 2020) and/or more targeted inspection of import goods from known source locations (Robinson et al. 2011), particularly in cases where the historical invasion pathway cannot otherwise be easily inferred. New genomic methods based on deep learning (e.g. Battey et al. 2020) have the ability to assign invasive species to their source location even under complex scenarios, especially when there is genetic similarity between the source and introduced ranges.

Genomic tools can help identify the mechanisms that allow invasive species to adapt to new environments or overcome stressors (see Genomic Signatures). In particular, theory suggests that species with lower genetic diversity may be less able to adapt and therefore easier to control, while those with high genetic diversity may be more adaptable/resilient and more challenging to manage. As well as quantifying diversity, genomic data can provide insights into the susceptibility of a species to management strategies, such as herbicides and/or pesticides, biocontrol agents, or genetic modification (Box 1). For example, cotton bollworm (*H. armigera*) is a global pest moth that recently invaded Brazil. Whole genome-resequencing data demonstrated that, shortly after the incursion, a pesticide resistance allele rapidly introgressed from *H. armigera* to the native corn earworm (*Helicoverpa zea*), highlighting required changes to the local management regime (Valencia-Montoya et al. 2020).

Box 1 **How genomic data can inform management outcomes**

**Fig. 4.**
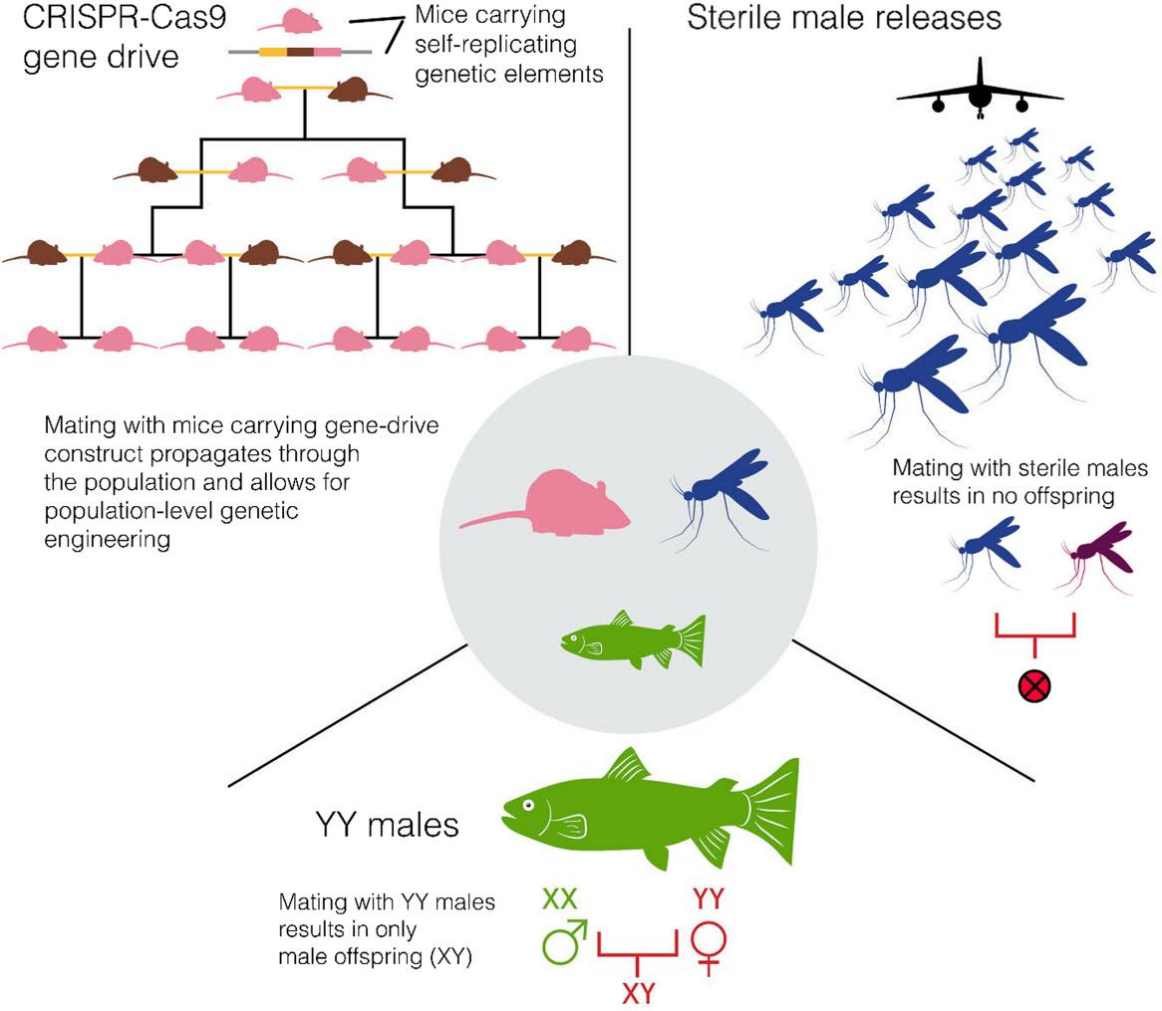
Genome-informed invasive species control tools.

Genome-informed population control technologies, such as the sterile insect technique, transgenic resistance, and gene drives can be implemented to manage invasive species (see Fig. 4).

Successful eradication of the invasive pink bollworm (*Pectinophora gossypiella*) is a testament to the sterile insect technique, with the population size of this highly invasive pest reduced to zero in Arizona in 2013 after reaching two billion individuals there in 2005 (Tabashnik et al. 2021). Negative growth rates were reported following each sterile insect release, and an estimated $200 million in savings followed eradication (Tabashnik et al. 2021).

Shifting the sex ratio of an invasive population toward a single sex is another genome-informed control technique; this one uses YY male or Trojan Y chromosomes. In the United States, this method is being used to control the invasive brook trout (*Salvelinus fontinalis*) and is predicted to eventually lead to population collapse—captive YY male broodstock are generated using exogenous sex hormone-induced sex reversal that produces YY-only sperm, resulting in sterile XY progeny upon mating with wild fish (Kennedy et al. 2018).

Another recent tool—the CRISPR-Cas9 gene-editing system—has hastened the development of gene drives (heritable elements that autonomously increase their frequency in the gene pool; Champer et al. 2016) targeting invasive species. Like the sterile insect technique, gene drives often include a sex-specific fitness cost that is introduced into an invasive population through releases of genetically modified individuals, but unlike sterile insect releases, they are self-propagating, leveraging the CRISPR-Cas9 system to bias their own inheritance. Gene drives have been demonstrated in a number of invasive insects, such as mosquitoes (*Anopheles gambiae* and *Aedes aegypti*; Hammond et al. 2016; Li et al. 2020) and moths (*Plutella xylostella*; Xu et al. 2022). Their use has also been demonstrated via transgenic generation and in silico population modeling in vertebrates, such as mice (*Mus musculus*), and their potential for the control of invasive vertebrates has been explored (Gierus et al 2022). Increasingly, these tools are becoming safer in some systems, with higher specificity and spatial confinement reducing untargeted impacts (Xu et al. 2022). However, social perceptions, local regulations, and concerns about containment limit their current development and deployment (Esvelt et al. 2014).

## Where Are We Heading?

Having outlined current approaches in invasion genomics, with a special focus on their ability to identify genomic signatures, predict invasive potential, and exploit new technologies for detection and biosurveillance, we now ask where the field is heading. During the recent inaugural conference on Invasion Genomics (November 2022; Aotearoa New Zealand), we developed a list of pressing questions that represent the next frontier of invasion genomics. Here, we first outline those questions, categorizing them into the paper's three broad foci, and we then highlight four key targets to prioritize.

### Genomic Signatures

- Are there broad trends in how the genomics of adaptation (i.e. diversity, genomic architecture, structural variation, and transposable elements) drives invasion success, or are species idiosyncratic? Evidence to date suggests the latter (Stern and Lee 2020; Pélissié et al. 2021), but what data would support a new invasion genomics paradigm?
- How do demography and genetic load affect invasion? Temporal impacts of such factors have been identified in small-population species conservation, but how they play out for invasive species remains unclear.
- What role does epigenetics play in invasion success? Epimutations rapidly emerge in response to environmental stress and thus likely play a role in a species’ ability to persist in new environments (Mounger et al. 2021). Analyzing epigenetic patterns in historical samples could date epimutations relative to DNA mutations and link them to trait variation and temporal changes in signatures of selection.

### Predicting Invasive Potential

- What makes a population invasive? Are there specific changes in genetic architecture or regions of the genome that predict invasiveness in related groups of taxa, is it dependent on phenotypic traits, or is it ecologically stochastic? Can we identify and integrate invasiveness traits that have a genomic basis and vary predictably across successful invaders into predictive models?
- Can we more frequently transfer lessons from invasion genomics to other fields? For example, greater integration between invasion biology and species conservation could improve understanding of the evolvability of invasive species and the drivers of successful population growth, including under changing climate conditions.
- By identifying resilient genotypes under certain environmental conditions, can we better predict the future direction of geographic range extension in invasive species?

### Technologies and Applications

- Can we improve the efficiency and speed of large-scale biosecurity methods (and the associated analytical tools), such as eDNA surveillance—especially in a rapid, real-time capacity? For example, incorporating machine learning digital scanning technology with genetics/genomics may revolutionize taxonomy and improve diagnostics by uniting robotics and artificial intelligence with molecular taxonomic identification (Srivathsan et al. 2021; Wührl et al. 2022).
- How can we increase the uptake of genomic tools and information in decision-making frameworks pertaining to invasive species management? Is this constrained primarily by funding limitations or also by social perceptions and local policy and governance?

### Future Targets

- Establishment of a worldwide invasion detection network that facilitates global collaboration, interdisciplinarity, and rapid response, coupled with standardization of national and international infrastructures for sample collection, storage, and data sharing (Box 2).
- Sequencing initiatives for a wide range of invasive taxa from native and expanded ranges, including generation of full genome and metabarcoding reference libraries.
- Genome-informed proactive management of invasive species, enabled by predictive understanding of invasion success—using high-resolution comparative genomics to identify genomic correlates of invasion success and predict invasiveness before establishment.
- Going beyond singular invasions to consider the whole community effects of invasion (i.e. “ecosystem homogenization”).

Box 2 **Data and networking recommendations**

Here, we evaluate the role of data sovereignty, metadata stewardship, research networking, and communication in invasion genomics and provide recommendations for the future.

**Data Sovereignty**

A typical requirement of genomics research is the deposition of raw sequence data in public archives/databases, and invasion genomics is no exception. Reference databases are essential for species identification and also serve as a source for molecular assay development. However, public accessibility of data is at times at odds with data sovereignty, which provides a way for Indigenous communities to take control of their data and share equitably in the benefits derived from it (Carroll et al. 2020; Friso et al. 2020).

Invasions are underpinned by the movement of species to novel environments, sometimes from native environments where they may be rare, treasured, or threatened. Thus, social contexts are important to consider (Yletyinen et al. 2021), especially when invasive species research involves collecting data from developing countries that may not have a well-resourced research community (Erondu et al. 2021). In such cases, the sharing of benefits derived from genetic resources may not occur and the local knowledge and research contributions from Indigenous people, local communities, and local scientists may fail to be acknowledged (Kukutai and Taylor 2016; von der Heyden 2023).

Researchers in invasion genomics science can ensure that they incorporate best practice approaches when generating genetic resources by (i) involving local scientists and, where possible, local communities to develop mutually beneficial outcomes and relationships that endure beyond interpreting and publishing the research (Wilcox et al. 2008); (ii) utilizing local infrastructure and collaborating with local institutions to support these communities and their development of capacity and capability; (iii) acknowledging resource and knowledge sharing in authorship where appropriate; and (iv) finding adequate compensation measures for Indigenous and local communities (whose livelihood, customs, or traditions may suffer as a consequence of control or management efforts) when developing invasive species management programs.

**Metadata Principles**

Embedded within the concept of data sovereignty are the FAIR (Wilkinson et al. 2016) and the complementary CARE principles (Carroll et al. 2020) of data stewardship. For effective analysis of invasive species genomic data, we require linkage of data at different levels (i.e. genome, transcriptome, and microbiome) across spatiotemporal trends (pre/postinvasion from native/expanded range across historical/contemporary boundaries), with findable connections between data resources. This requires all parties to have followed FAIR guiding principles to make data accessible, in addition to consultation with Indigenous and local groups on data stewardship and collectively beneficial outcomes.

Standardized reporting of all available metadata for genomic data from both historical and contemporary samples will provide an ongoing resource for researchers beyond the scope of the initial research—especially because recoverability of “missing” metadata is low (Crandall et al. 2023). When capturing sample and metadata information, an agreed set of uniform language and required fields should be integrated, placing high importance on data provision associated with habitat, host, and native/invasive status (Vaughan et al. 2023). Standardized bioinformatic pipelines, from processing raw sequencing data to quality control and analysis, would also increase usability and comparisons across studies. This could be facilitated by the incorporation of minimum metadata standards to enforce uniformity and reproducibility—for example, via consistent metadata and code availability standards by journals. The recent establishment of several initiatives (e.g. Earth Biogenome Project, https://www.earthbiogenome.org; Darwin Tree of Life, https://www.darwintreeoflife.org) provides excellent examples that support ethical and legally compliant sample and metadata collection (Formenti et al. 2022).

**Networking**

Knowledge transfer is an integral part of facilitating efficient and inclusive research, whether by sharing data across platforms, building individual researcher capability by training, or providing logistic support. For example, the generation of genomic data by developing countries can be restricted by limited expertise and resources—including access to storage and server space for genomic analyses. To combat this, cross-country collaboration agreements that provide equitable access to resources can be established. If a centralized process for training and data sharing is in place, a further benefit is that data can become comparable on a global scale to facilitate novel outcomes. For example, initiatives such as MalariaGen (https://www.malariagen.net) offer centralized training and place strong emphasis on capacity and capability building, while the European Reference Genome Atlas (a pan-European approach to providing reference genomes; Formenti et al. 2022) is paving the way in the distribution of facility networks.

**Effective Scientific Communication**

In addition to interinstitutional collaboration, democratic approaches to invasive species management must include public participation, especially where the response to public concern can greatly influence the policy of management strategies (Crowley et al. 2017). A crucial element that promotes the successful involvement of individuals and communities is clear and effective science communication. When this is done well, a major benefit is the incorporation of citizen science in invasive research. For example, in Aotearoa New Zealand, national campaigns are commonly employed by the Ministry for Primary Industries to encourage the rapid reporting of invasive pest sightings by the general public that can help to curb their spread. Other countries with high levels of endemism and a similar island-based border (Veron et al. 2019) could deploy similar levels of government response to help build public interest and active participation in invasive species detection (e.g. https://www.iucncongress2020.org/motion/116).

## Conclusions

As contemporary climate change and biological invasion jointly compound their impacts on native biodiversity globally, the use of novel genome-informed tools for targeted management and mitigation needs to grow. While recent technological advances are improving knowledge of how genome evolution can facilitate invasion, there is still much to learn. Here, based on international expert opinion during the 2022 inaugural conference on Invasion Genomics, we have outlined key advances and set the agenda toward future research objectives that put the genome at the center of invasion research. Among these, perhaps the most important is that we draw together genome-informed understanding and tools to create a more comprehensive response to biological invasion that is more proactive than reactive. A proactive approach will require continued genomic research into the mechanisms and pathways of successful and failed invasions, and the leveraging of this information into the development of tools that can slow spread, mitigate impacts, and/or support eradication campaigns for destructive invasive species. Implementing complementary infrastructure for data and metadata ownership, sharing, storage, and communication among the global genomics community will best prepare us for the future.

**Glossary of Terms**

- **Balanced polymorphism—**The maintenance of multiple alleles in a genomic region through balancing selection. This can occur because the marginal fitness of alleles differs over space or time or when heterozygotes have higher fitness than homozygotes.
- **Balancing selection—**A form of natural selection that maintains genetic variation within a population as multiple alleles at a specific locus and occurs when different alleles are favored in different environments or at different points in time, or when heterozygotes have a fitness advantage over homozygotes.
- **Bridgehead effect**—When successful invasions involve a particular invasive population that serves as the source of additional invasions via secondary introductions (i.e. “invasion begets invasion”).
- **CARE principles—**The CARE principles for Indigenous Data Governance complement the FAIR principles, encouraging open data, considering people and purpose, and encompassing consideration of Collective benefit, Authority to control, Responsibility, and Ethics.
- **CRISPR-Cas9—**CRISPR (clustered regularly interspaced short palindromic repeats) are specific DNA sequences found in prokaryotic genomes that, when combined with RNA-guided endonuclease enzymes (such as Cas9) that can cut DNA at a desired location within the genome, can be used as a gene-editing tool.
- **Demographic swamping—**A process by which sterile hybrids, or those of reduced fitness, decrease the population growth rate.
- **Directional selection—**A form of natural selection that favors individuals with extreme phenotypes, causes a shift in allele frequency toward that phenotype over successive generations, and reduces genetic diversity at the selected and linked loci.
- **DNA metabarcoding**—High-throughput sequencing of DNA extracted from an environmental sample, where DNA is amplified by a polymerase chain reaction (PCR) using primers that target multiple taxa. The use of a large number of unique barcodes enables the simultaneous identification of multiple taxa in a single sample.
- **Environmental DNA (eDNA)**—DNA extracted from environmental samples, including water, soil, and air.
- **FAIR principles—**The FAIR guiding principles of data stewardship determine that both data and its associated metadata should be Findable, Accessible, Interoperable, and Reusable.
- **Founder effect**—A reduction in genetic variation that results when a small number of individuals from a larger population establish a new population.
- **Genetic admixture—**The presence of genetic variants from at least two genetically differentiated populations in one or more individuals as a result of hybridization.
- **Genetic swamping—**Any process by which alleles characteristic of a native population are replaced by those from a nonnative population.
- **Hybridization—**Reproduction between individuals of genetically differentiated populations (which may include different species), resulting in offspring with mixed genetic ancestries.
- **Introgression—**Transfer of an allele from one genetically differentiated population to another via repeated hybridization and backcrossing. This may include different species.
- **Phenotypic plasticity—**The ability of an organism to exhibit different phenotypes from the same genotype in response to changes in its environmental conditions.
- **Polyploidy—**The heritable condition of possessing more than two complete sets of chromosomes.
- **Quantitative polymerase chain reaction (qPCR)**—a PCR method that quantifies the amount of starting DNA or RNA using fluorescence probes or dyes.
- **Secondary contact—**The process by which previously isolated populations come back into contact with each other.
- **Standing genetic variation—**The presence of more than one allele at a locus in a given population. Such variation can arise due to gene flow, mutation, and recombination events and serves as the raw material for evolutionary processes, such as natural selection.

## Data Availability

No new data were generated as part of this review.
